# Social Network Correlates of Mental Health among Rural-to-Urban Migrants in China

**DOI:** 10.3390/ijerph182010902

**Published:** 2021-10-17

**Authors:** Wenqing Wang

**Affiliations:** School of Humanities and Social Sciences, Beijing Institute of Technology, Beijing 100081, China; wenqingwang@bit.edu.cn; Tel.: +86-10-8138-1301

**Keywords:** migrant worker, rural-to-urban migrant, mental health, social network, inter-hukou network, social support, weak ties, China

## Abstract

Internal rural-to-urban migrants in China are facing a high risk of mental disorders. Previous research on mental health correlates and predictors among this population focused on individual-level characteristics, neglecting network-level indicators, and migrant–urbanite intergroup relationship. A cross-sectional survey was conducted in Beijing, China from December 2018 to January 2019. A convenience sample of 420 rural-to-urban migrants completed the Chinese 12-item General Health Questionnaire and reported their relationship with urbanites in the past six months. Multivariate linear regression models were used to test the association of the inter-hukou network with migrant mental health. Two indicators of the inter-hukou network were significantly associated with migrant mental health. Migrants were more mentally healthy if their proportion of weak ties in the inter-hukou network was no less than 50%. The more social support migrants received from the inter-hukou network, the better their mental health was. Meanwhile, there was a significant interaction effect between social support and sex, indicating that the same level of social support better protected the mental health of female migrants. Results suggest the importance of social network factors and migrant–urbanite ties for migrant mental health. Future efforts may need to mobilize and facilitate the inter-hukou network to improve migrant mental health.

## 1. Introduction

A series of suicides committed by more than ten rural-to-urban migrant workers in a factory around 2010 in Shenzhen, China rang the alarm for the poor mental health of the millions of Chinese migrant workers and spurred the research on the subject [1]. Recent studies showed that mental health problems were quite common among migrant workers. A cross-sectional survey conducted in two shoe-making factories in China found that 31.7% of 915 assembly-line migrant workers were clinically depressed [2]. The prevalence of common mental health problems was 34.4% in a random sample of 3031 migrant workers of ten manufacturing factories in Shenzhen, China [3]. Compared to the residents with urban hukou, rural-to-urban migrants were facing a higher risk of mental health [4].

More than a century ago, *Suicide* by Durkheim revealed that social relations could exert significant effect on the wellbeing of individuals [5]. In recent decades, vast research on social support [6], social groups [7] (p. 115), and social networks [8] (pp. 293–322) further demonstrates the significance of social relations and social networks to mental health. Thus, it is necessary to ask what roles social relations and social networks play in migrant mental health. Apparently, in the daily life of rural-to-urban migrants, their relationship with local urbanites—local residents with urban hukou—constitutes an important part of their social environment. Then, what effect does the migrant–urbanite intergroup network exert on migrant mental health?

Based on the field work conducted in ‘*Zhejiangcun*’—a migrant settlement in Beijing —in late 1990s, Zhang described rural-to-urban migrants as ‘strangers in the city’, which epitomizes the scarce and superficial interaction between migrants and urbanites, the marginalization of migrants in cities, and the discrimination and social exclusion against migrants. The organization of space played an important role in the process. As a peculiar kind of migrant residential space inhabited exclusively by migrant businessmen and their workers, *dayuan* (which can be roughly translated as ‘big yard’ or ‘big compound’) facilitated the internal interaction among migrants but impeded the interaction between migrants and urbanites [9] (pp. 69–90). Jacka, who conducted field work in Beijing at roughly the same time, also found that migrant women rarely made friends with local urbanites, that their experiences with urbanites were normally negative, and that there was almost no shared interest and sense of belonging between two groups [10] (pp. 201–202). In a word, around the year of 2000, the social segregation of migrants led to the ‘dual community’ in cities of China [11]. The negative intergroup relationship might have a bad influence on migrant mental health, but at that time, such influence rarely drew attention from the academic community.

Twenty years since then have witnessed changes in the migrant–urbanite relationship. The intergroup interaction has moved from ‘dual community’ to ‘befriending the other’ [12]. Migrants began to make deeper associations with local urbanites, and some of them even made friends with urbanites [13]. From the perspective of social network, dual community means that urban social network was divided into two relatively separate parts—migrant network and urbanite network, and the connections between them were sparse and weak. The transition from dual community to befriending the other would then imply that various bridging connections between migrant network and urbanite network gradually emerge, and urbanites become an increasingly important part in the ego-centered network of migrants. If this is the case, does the addition of urbanites to migrant social network bring benefits to migrant mental health?

To answer the question, the newly developed part of migrant network has to be measured in advance as an independent construct. Unfortunately, however, although research on migrant social network flourished in China in the past several decades, most research did not differentiate urbanites from other members in network measurement. Therefore, what they explored was merely ‘*general* social network’ of migrants. Efforts, though still very limited, began to emerge recently to tease out a separate network by asking migrant participants to report on urbanite network members. For reasons of both convenience and precision, this new network may be termed as an inter-hukou network of migrants given that it crosses the boundary set by the official household registration system (hukou system) and connects rural hukou holder with urban hukou holder.

For example, researches have investigated the role of social networks in the integration of Chinese rural–urban migrants from a ‘migrant–resident tie’ perspective [14]. Survey respondents were asked to report “Over the past few months, due to private matters rather than work issues, how many family members or relatives, and friends have you contacted frequently with face-to-face meetings, phone or text messages, or email/snail mail”. They were then asked to report the specific number of local urban residents among relevant nodes. This approach to operationalizing migrant–resident ties was adapted from the ‘New-Year-Greeting networks’ specifically designed for Chinese [15], with the time frame in the question being changed from new year festival to the past few months before the survey.

Attention to the role of the inter-hukou network was more recent in the research on migrant mental health which has long focused on individual-level risk factors including socioeconomic factors [16], housing conditions [17], migration stress and social competence [18], generational factors [19], physical illness, infrequent hometown visit, multi-city migration experience, poor Mandarin proficiency, and working more than 8 hours per day [3]. When network-level variables were included in the analysis, it was more likely about general social network. For instance, using data from the Rural-to-Urban Migration in China (RUMiC) project, a study investigated the protective role of migrant social networks in host cities on mental health [20]. Here, migrant social network was a general one because nodes with and without urban hukou were mixed together. Interestingly, although the RUMiC project asked specific questions about nodes with urban hukou, and hence was able to construct an inter-hukou network for each migrant respondent, only the size of general social network was included in the analysis. Up to now, only one study was found to have included inter-hukou network measurement in the analysis of migrant mental health. Similar to efforts by Yue et al. [14] and the RUMiC project [20], the study drew on the ‘New-Year-Greeting networks’ approach [15] when it constructed an inter-hukou network for migrant respondents [21]. ‘Perceived social support from nodes with urban hukou’ in the study was actually measured by the size of inter-hukou network, which is the only inter-hukou network measurement in the study.

To summarize, little is known about the inter-hukou network of migrants and its effect on migrant mental health. In fact, an image of the inter-hukou network as a whole was still vague even when inter-hukou network measurements were included in analysis in rare cases. Highlights were either on nodes and social support from them [21] or ties between migrant and urbanite [14], while the network constituted by these nodes and ties barely got a proper name. The anonymity of the new network both resulted from and in a lack of attention. The efforts at naming, though seemingly superficial and insignificant, may make up one of the first steps in breaking the vicious circle of silence.

### Research Framework and Hypothesis

Although social relationships have a ‘dark side’ and can exert negative influence [22], this study approaches social relationships and social networks from a positive perspective and regards them as resources that can bring benefits to migrant mental health. Compared to other social networks of migrants, an inter-hukou network is irreplaceable in that it can provide special protection to migrant mental health.

First, the irreplaceability of the inter-hukou network lies in its ability to integrate migrants into urban society [14]. Of course, the intragroup social relationships among migrants also play an integrative role by reinforcing internal solidarity. However, as indicated by Zhang [9], internal solidarity among migrants could be barriers to intergroup connections between migrants and urbanites. The positive migrant–urbanite interaction is the inevitable path from “dual community” to “befriending the other” and then to the deep social integration of the whole urban population [12]. Thus, the emergence of the inter-hukou network is both the result of positive migrant–urbanite interaction and the indicator of urban social integration. It implies that migrants and urbanites have gradually developed personal relationships through which migrants experience their being accepted by urbanites, enhance their sense of belonging and self-esteem, and recognize the city as their home and themselves as urbanites. Since sense of belonging and self-esteem are among the mechanisms through which social relationships exert protective influence on mental health [23], we have good reason to believe that the existence of the inter-hukou network has a positive influence on migrant mental health, and that the bigger the size of the inter-hukou network, the more the protection there is.

**Hypothesis** **1.** *If migrants have a bigger size of inter-hukou network, their mental health is better.*

Second, the inter-hukou network holds not only more but unique resources which migrants cannot get from other social networks. Needless to say, in Chinese cities, urbanites have greater access to social resources, on average, because of their advantages in economic, political, and cultural fields [15]. Furthermore, some resources are unique to urbanites because of institutionalized discriminations against migrants, including the formal social security system based on hukou status and the informal differentiation in social identity, lifestyles, and moral values. On the one hand, institutionalized differences impede deep interaction and make it hard to develop intimate relationship between migrants and urbanites. On the other hand, once migrants break through the barriers and make intimate enough relationship with urbanites, they benefit from the unique resources from urbanites. It is well documented that social networks provide important channels for the flow of emotional, informational, and instrumental support [24], and social support is an important mechanism through which social network improves mental health [25]. If social resources from the inter-hukou network are conceptualized as social support, it is reasonable to suppose that they provide protection to migrant mental health.

**Hypothesis** **2.** *If migrants receive more social support from inter-hukou network, their mental health is better.*

The seminal work by Granovetter reveals the strength of weak ties [26], which reminds us that the positive influence of the inter-hukou network on migrant mental health may not result merely from strong ties. For example, the sense of belonging enhanced by weak ties also matters for mental health [23]. Furthermore, weak ties are in advantageous positions to provide health information. For one thing, health information conveyed by weak ties are regarded as more objective and effective because communications through weak ties are thought to be less emotional; for another, people perceive less stress when exposing their disease to weak ties because they worry less about being discriminated [27]. Besides, for people with special needs, although significant others usually have strong willingness to help, they may not know how to do because they have not experienced the disease for themselves. On the contrary, weak ties with similar disease experience can provide emotional support (e.g., sympathetic understanding and tolerance), pertinent information and advices (e.g., risk assessment and coping strategies), and role models [23]. Thus, it is reasonable to believe that weak ties in inter-hukou network can protect migrant mental health by promoting social integration and providing social support.

**Hypothesis** **3.** *If migrants have higher proportion of weak ties in inter-hukou network, their mental health is better.*

Past research has revealed the sex difference in the effect of social network on mental health [28]. For example, the loss of a marital partner resulted in a relatively greater increase in morbidity and mortality among widowers than among widows in the acute grieving period [29]. Another example is that social support from extended family had positive influence on men’s mental health but negative influence on women’s mental health [30,31]. If sex difference can be found in the influence of marriage and extended family on mental health, it may also be found in the influence of inter-hukou network on migrant mental health.

**Hypothesis** **4.** *There are interaction effects between sex and network indicators in the association of inter-hukou network with migrant mental health.*

## 2. Methods

### 2.1. Study Location and Sampling

The data were collected from December in 2018 to January in 2019 among rural-to-urban migrants in Beijing, China. Beijing was chosen as the research city for several reasons. First, as the capital of China and one of the several super cities in China, Beijing attracted many migrants. In 2019, Beijing had a population of 21.54 million, 35% (7.57 million) of whom were migrants [32]. Second, migrants in Beijing were more socially excluded because the household registration system in Beijing was stricter and it was more difficult for migrants to change their hukou status from rural residents to urban residents, which makes it more necessary to investigate inter-hukou network and its effect on migrant mental health. Finally, Beijing Social Science Foundation, i.e., the funder of this research, prioritizes research focusing on Beijing.

In this article, a migrant is defined as a person who lives in Beijing but has a rural *hukou* (official household registration status) outside of Beijing. Two urban districts and one suburban district in Beijing were chosen as the sites for sampling. The inclusion criteria of participants were as follows: (a) aged 18 to 65 years old; (b) registered as a rural resident outside of Beijing; and (c) lived in Beijing for six months at least. It is easier to obtain a random sample of migrants in places such as factories where a full list of migrants is available [3]. However, in open social spaces where migrants mix together with local residents, it is much more difficult to gain a random sample of migrants because many migrants are not registered officially and a good enough sampling frame is not available. Convenience sampling was thus used to recruit migrants in this research. Nevertheless, in order to increase the heterogeneity of the sample, efforts were made to include all the typical migrant occupations and keep a sex balance. In places where migrants work—such as construction sites, factories, markets, and restaurants—interviewers approached them to confirm their migrant status, introduce the survey, and invite them to participate. The research instrument was a self-administered questionnaire, with both printed copy and digital version for participants to choose. The digital questionnaire was stored on an Internet server, and accessed and delivered through mobile phones or tablets connected to the Internet.

Five hundred and thirty migrants consented to participate in the survey, 110 of whom were excluded from the dataset for final analysis. Twelve participants were excluded because they selected the “Urban Hukou” option. Sixteen participants were excluded because of the omission of key questions. In addition, eighty-two participants whose size of inter-hukou network was zero were excluded because the zero size of network would make other two network indicators—social support and the proportion of weak ties—meaningless. Therefore, the sample size for final data analysis was 420 participants.

Participants received 50 RMB (about 7 USD) remuneration. All procedures performed in this study were in accordance with the 1964 Helsinki declaration and its later amendments or comparable ethical standards. Ethical approval was obtained from the ethics committee of the Beijing Institute of Technology.

### 2.2. Measurement

#### 2.2.1. Dependent Variable

The dependent variable in this research is mental health, measured by the General Health Questionnaire (abbreviated thereafter as GHQ), which is widely used to screen general (non-psychotic) mental health problems. GHQ was originally developed as a 60-item instrument, but now there are various shortened versions, including the GHQ-30, the GHQ-28, the GHQ-20, and the GHQ-12, among which the GHQ-12 is the most popular because of its simplicity [33]. This research employed the Chinese version of the GHQ-12, whose validity and reliability had been established by past research [34]. The Cronbach’s Alpha of the questionnaire in this research is 0.87.

The Chinese GHQ-12 consists of 12 items, including 6 positively worded items and 6 negatively worded items. Each item aims to assess the severity of a mental problem over the past few weeks. A 4-point Likert-type scale (from 0 to 3) was used to rate the items. The positive items were corrected from 0 (always) to 3 (never) and the negative ones from 3 (always) to 0 (never). The score was used to generate a total score ranging from 0 to 36, with higher scores indicating worse health. Scores over the cut-off point of 11/12 could be classified as cases demanding special attention [35,36].

#### 2.2.2. Independent Variable

Independent variables are the three indicators of the inter-hukou network: network size, the proportion of weak ties, and social support. Drawing on the study by Yue et al. [14], migrant respondents were asked to report “Over the past six months, due to private matters rather than work issues, how many urbanites (local residents with urban hukou) of the following three categories have you contacted frequently with face-to-face meetings, phone or text messages, or email/snail mail? (1) Urbanite relatives (urbanites who are your relatives); (2) Urbanite friends (urbanites who are your friends, excluding urbanite relatives); (3) Urbanite acquaintances (urbanites who are your acquaintances, excluding urbanite relatives).” Respondents were asked to report the number of each category, respectively. The size of inter-hukou network was calculated by summing the three numbers above.

There are several methods of measuring tie strength, including, but not limited to, relationship category, interaction, and the multi-indicator method [37]. In order not to pose a significant data-reporting burden on migrant participants who have extremely limited time to contribute to research studies, this research employed the simple and convenient method of relationship category, treating relatives and friends as strong ties and acquaintances as weak ties. Thus, the proportion of weak ties meant the proportion of urbanite acquaintances in all the ties of inter-hukou network. In the statistical analysis, the proportion of weak ties was transformed into a binary variable, with the cut-off point being 50%, ≥50% being 1, and <50% being 0.

Social support was measured with an 8-item scale (Cronbach’s Alpha 0.85), which captured the emotional support (e.g., urbanites provide comfort when you are in bad mood), information support (e.g., urbanites discuss important issues in your life with you), social companionship (e.g., urbanites join you in leisure and recreation activities), and instrumental support (e.g., urbanites lend money to you). Each response was coded on a three-point scale from “often” (2 points) to “seldom” (1 point) and to “never” (0 point). A composite score was calculated by adding responses of the eight items (range 0–16), where higher composite scores indicate more social support migrant participants received from urbanites.

#### 2.2.3. Control Variables

To better verify the influence of inter-hukou network on migrant mental health, variables that may affect mental health were introduced into statistical analysis as controls, which include sociodemographic characteristics (e.g., age, sex, marital status), migration characteristics (e.g., number of jobs ever pursued in Beijing, frequency of visiting hometown), work environment (e.g., health threat, medical insurance), housing environment (e.g., housing facilities, neighborhood composition), and physical health condition (physical discomfort).

### 2.3. Statistical Analysis

Although mental health scores obtained from psychological scales are essentially ordinal, they are usually treated as interval variable in statistical analysis. Following the tradition, multivariate linear regression was used in this research. All the independent and control variables were included in the first model. Three interaction terms between sex and three independent variables were further introduced in the second model. Variable centering was employed in computing interaction terms to avoid multicollinearity. Categorical variables were transformed into dummy variables before being included in the models. The results of statistical test showed that multicollinearity was not a problem for both models.

## 3. Result

### 3.1. Sociodemographic and Network Characteristics

Sociodemographic and network characteristics of participants are reported in Table 1. The mean score of mental health for the sample (M = 14.28, SD = 4.49) surpassed the cut-off score on the GHQ-12 and indicated the poor mental health of migrant participants. The mean size of the inter-hukou network (M = 11.54, SD = 16.75) revealed that urbanites had become an unignorable part of migrant social network. Half of participants reported that their proportion of weak ties was no less than 50%. The mean score of social support (M = 5.51, SD = 3.94) was slightly above one third of the possible range and indicated that urbanite social support was still quite limited.

### 3.2. The Effect of the Inter-Hukou Network on Mental Health

Table 2 presents the results of multivariate linear regression analysis of the effect of inter-hukou network on migrant mental health. In Model 1, the coefficient of the size of inter-hukou network was negative (B = −0.008, SE = 0.014) but insignificant, which indicates that hypothesis 1 was not supported although the size of inter-hukou network had a protective effect on migrant mental health. Compared to migrants whose proportion of weak ties was below 50%, the mental health scores dropped by 1 point on average for migrants whose proportion of weak ties was no less than 50% (B = −0.938, SE = 0.437, *p* < 0.05), which means that higher proportions of weak ties provided protection for mental health. Thus, hypothesis 2 was supported. With each one-point increase in social support score, mental health scores of migrants decreased by 0.139 points (B = −0.139, SE = 0.060, *p* < 0.05), indicating that social support from urbanites could also provide protection for migrant mental health. So, hypothesis 3 was supported, too. Among the control variables, a monthly income of more than 5000 yuan (B = −0.869, SE = 0.478, *p* < 0.1), blue-collar worker (B = −1.492, SE = 0.795, *p* < 0.1), and shopkeeper/contractor (B = −1.837, SE = 0.921, *p* < 0.05) had a protective effect on the mental health, while the number of jobs pursued in Beijing (B = 0.238, SE = 0.085, *p* < 0.01), lived in neighborhood with more migrants than urbanites (B = 0.832, SE = 0.441, *p* < 0.1), and physical discomfort in past 3 months (B = 1.669, SE = 0.438, *p* < 0.001) increased the risk of mental health problems.

Three interaction terms between sex and independent variables were added into model 2, but only the interaction between sex and social support was significant (B = −0.276, SE = 0.119, *p* < 0.05). With social support being equal, the mental health scores of female migrants were lower than those of male migrants by 0.276 point on average. These results partly supported hypothesis 4. It was noteworthy that, after introducing interaction terms, the effect of social support on mental health was still significant and the significance level changed little (B = −0.128, SE = 0.060, *p* < 0.05). It follows that main effect and interaction effect both existed in the influence of social support on mental health. The protection of higher proportion of weak ties on mental health was also still significant. Among the control variables, their significance remained on the same level except for shopkeeper/contractors.

## 4. Discussion

This study examined the association of the inter-hukou network with migrant mental health and found that two indicators of the inter-hukou network were of significance. Migrants were more mentally healthy if their proportion of weak ties in the inter-hukou network was no less than 50%. The more social support migrants received from the inter-hukou network, the better their mental health was. Furthermore, the same level of social support brought more benefits to the mental health of female migrants.

Ever since the classical study by Granovetter [26], lots of attention has been given to weak ties whose significance to information transmission and job opportunity has been widely confirmed. However, the effect of weak ties on mental health rarely drew attention, and limited existing research revealed that weak ties had a negative influence on mental health. For example, loose networks dominated by weak ties were found to bring benefits to physical health but harms to mental health; in interpreting the result, the author supposed that dense networks are more important to mental health because it depends more on expressive actions, and that loose networks are more important to physical health because it depends more on instrumental actions [38]. In other words, previous research tended to believe that weak ties facilitate instrumental actions well but do a poor job in meeting emotional needs and in improving mental health. Recent research, however, suggested that there are at least two ways through which weak ties can bring benefits to mental health. One is that the instrumental action facilitated by weak ties directly contributes to the coping with psychological stress (e.g., enhancing risk appraisal by providing more objective information). The other is that weak ties may satisfy some emotional needs which cannot be met by strong ties (e.g., compared to strong ties without certain experience, weak ties who have similar experience with a recipient are easier to convey sympathy, understanding, and tolerance) [23]. Of course, further exploration and verification are needed to find out whether the two mechanisms can explain the protective effect of weak ties on migrant mental health.

In this article, the proportion of weak ties plus that of strong ties is equal to 1. Therefore, as a relative measurement, the proportion of weak ties reflected the constitution of the inter-hukou network and could be easily translated into the proportion of strong ties. For example, the result that migrants were more mentally healthy if their proportion of weak ties in inter-hukou network was no less than 50% can be rephrased as follows: migrants were less mentally healthy if their proportion of strong ties in the inter-hukou network was more than 50%. It is thus clear that, although strong ties can usually provide stronger emotional support and psychological assistance, more strong ties do not necessarily lead to better mental health. When the proportion of strong ties exceeds a certain limit (e.g., 50% in this article), the negative impact on mental health might arise. In this sense, the key for a better mental health may lie in the relative constitution of strong and weak ties rather than the absolute number of strong ties.

There is an abundant literature on the protective effect of social support on mental health. A few studies also revealed that social support reduced mental health risk for rural-to-urban migrants in China [39,40]. However, in measuring social support, almost no research clarified whether support providers were urbanites (local residents with urban hukou). This research is among the first, if not the first, to indicate the significance of urbanite social support to migrant mental health. Another point worthy of noting is that this research investigated received social support as opposed to perceived support. The literature on social support often differentiates perceived social support from received support because usually there is only a weak correlation between them, with the effect of the former being stronger and more consistent and that of the latter weaker and contradictory [41,42]. More research needs to be carried out to see if the result of this research can be replicated in the future.

The finding that same level of social support from the inter-hukou network brought more protection to the mental health of female migrants echoes past research by confirming the sex difference in the social network effect on mental health [28]. With regard to which sex benefits more, however, no unitary picture was disclosed by previous research. For example, an early research found that extended kin support was associated with fewer symptoms of depression, but such support was less beneficial for females in general and not beneficial at all for females aged 17 to 34 in particular [30]. In contrast, a recent research indicated that female mental health appeared to benefit slightly more from higher levels of social support than males [43]. The result of this research is more in line with the latter one. These contradictory results make further explanations both difficult and necessary. In explaining the paradoxical finding that young women perceived the highest level of extended kin support and yet reported the greatest number of depressive symptoms, Dressler emphasized the ‘psychological cost’ of extended kin support in a traditional and conservative community where young women came under closer scrutiny and were expected to follow more closely the advice of their (usually older) support system members [30]. From this viewpoint, we may suppose that the mental health of female migrants benefited more from urbanite support because they experienced less psychological costs than their male counterparts. Future research is needed to determine whether the supposition can hold up under testing.

The size of the inter-hukou network was not associated with migrant mental health, which is not consistent with some previous research [21] but fits well into the general picture of mixed results regarding the correlation between network size and mental health [44]. The complexity of the correlation was manifested very clearly in a study conducted among college students in the US which revealed that greater network size, while significantly reducing risk of depression among students of Asian/Pacific descent, had no significant effect on anxiety among the same ethnic group and had no significant effect on both depression and anxiety among Caucasian students [45]. These results suggest that future research should pay more attention to the complex mediating mechanisms underlying the relation between network size and mental health.

There were several limitations to this study. First, a cross-sectional design limited our ability to make a causal inference of the relations between network variables and mental health. Second, a convenience sample might limit the generalizability of our findings. However, we made efforts to increase the heterogeneity of the sample by including all the typical migrant occupations and keeping a sex balance. Participants were sampled from diverse recruitment settings which resulted in greater heterogeneity across demographics and socioeconomic strata of migrants. Nevertheless, future studies employing a random sampling are necessarily called for to verify the results of this study. Third, inter-city comparison was not conducted in this research because of limited budget. Future research may need to include diverse cities and make comparison between them, considering that the association of the inter-hukou network with migrant mental health might be different in cities of different sizes and/or of different administrative rankings (e.g., national capital city vs. county town). Lastly, migrant social networks other than the inter-hukou network were neither separately measured nor controlled in statistical analysis, which makes it hard to differentiate the contribution of the inter-hukou network to migrant mental health from the contribution of other migrant social networks. To better verify the independent effect of the inter-hukou network on migrant mental health, future efforts need to take into consideration different social networks and their effects simultaneously.

With the Likert scoring methods, GHQ-12 scores over the cut-off point of 11/12 can be classified as cases demanding special attention [35,36]. In this migrant sample, the mean GHQ-12 score was 14.28 points and the scores of 75% of migrants were 12 points or above, which was consistent with previous research findings that mental health risks among rural-to-urban migrants were worrisome [2,3]. This situation merits more attention from researchers and policy makers alike. Since inter-hukou network is likely to provide protection to migrant mental health, future social policy should promote social interaction between migrants and urbanites, improve the migrant–urbanite relationship, and stimulate the development of inter-hukou network.

## 5. Conclusions

This study examined the association of the inter-hukou network with migrant mental health. Results suggest that internal rural-to-urban migrants in China are facing high risk of mental disorders, and that social network factors and migrant–urbanite ties are of significance for migrant mental health. Future efforts may need to mobilize and facilitate the inter-hukou network to improve migrant mental health.

## Figures and Tables

**Table 1:** Sociodemographic and network characteristics of participants (*n* = 420).

Characteristics	*n* (%)
Age *	31.10 (9.59)
Sex	
Male	277 (65.95)
Female	143 (34.05)
Marital status	
Married	228 (54.29)
Other	192 (45.71)
Education	
≥High school	240 (57.14)
<High school	180 (42.86)
Monthly income	
>5000 yuan	160 (38.10)
≤5000 yuan	260 (61.90)
Occupation	
Blue-collar worker	278 (66.19)
Stall vendor	47 (11.19)
Shopkeeper/contractor	58 (13.81)
Other	37 (8.81)
Jobs ever pursued in Beijing *	2.62 (2.51)
Frequency of visiting hometown	
≤once a year	218 (51.90)
>once a year	202 (48.10)
Health threat in work environment	
Yes	146 (34.76)
No	274 (65.24)
Medical insurance coverage	
No	92 (21.90)
Yes	328 (78.10)
Housing facility	
Bad	82 (19.52)
Good	338 (80.48)
Neighborhood	
Migrants > urbanites	204 (48.57)
Migrants ≤ urbanites	216 (51.43)
Physical discomfort in past 3 months	
Yes	192 (45.71)
No	228 (54.29)
Mental health *	14.28 (4.49)
Size of inter-hukou network *	11.54 (16.75)
Proportion of weak ties	
≥50%	224 (53.33)
<50%	196 (46.67)
Social support *	5.51 (3.94)

* Mean (SD).

**Table 2:** Multivariate linear regression analysis of the association of the inter-hukou network with mental health.

	B (SE)	
	Model 1	Model 2
Age	0.018 (0.029)	0.015 (0.030)
Sex (female = 1)	0.638 (0.468)	0.621 (0.468)
Marriage status (married = 1)	−0.546 (0.571)	−0.502 (0.571)
Education (≥high school = 1)	−0.027 (0.471)	0.079 (0.473)
Monthly income (>¥5000 = 1)	−0.869 (0.478) +	−0.924 (0.479) +
Blue-collar worker (yes = 1)	−1.492 (0.795) +	−1.405 (0.794) +
Stall vendor (yes = 1)	−1.039 (1.021)	−1.046 (1.018)
Shopkeeper/contractor (yes = 1)	−1.837 (0.921) *	−1.697 (0.921) +
Jobs ever pursued in Beijing	0.238 (0.085) **	0.244 (0.085) **
Frequency of visiting hometown (≤once a year = 1)	0.276 (0.425)	0.209 (0.425)
Health threat in work environment (yes = 1)	0.693 (0.476)	0.613 (0.476)
Medical insurance coverage (no = 1)	0.052 (0.529)	0.197 (0.532)
Housing facility (bad = 1)	0.625 (0.566)	0.581 (0.565)
Neighborhood (Migrants> urbanites = 1)	0.832 (0.441) +	0.754 (0.443) +
Physical discomfort in past 3 months (yes = 1)	1.669 (0.438) ***	1.660 (0.437) ***
Size of inter-hukou network	−0.008 (0.014)	−0.009 (0.014)
Proportion of weak ties (≥50% = 1)	−0.938 (0.437) *	−0.949 (0.437) *
Social support	−0.139 (0.060) *	−0.128 (0.060) *
Size of inter-hukou network × female		0.034 (0.028)
Proportion of weak ties × female		−0.311 (0.909)
Social support × female		−0.276 (0.119) *
Constant	13.417 (1.254) ***	13.420 (1.254) ***
R^2^	0.137	0.149
F	3.539 ***	3.323 ***
*n*	420	420

+ *p* < 0.1; * *p* < 0.05; ** *p* < 0.01; *** *p* < 0.001.

## Data Availability

The data presented in this study are available on request from the corresponding author. The data are not publicly available due to restrictions set by the ethical committee.

## References

[1] Lau J.T., Cheng Y., Gu J., Zhou R., Yu C., Holroyd E., Yeung N.C. (2012). Suicides in a mega-size factory in China: Poor mental health among young migrant workers in China. Occup. Environ. Med..

[2] Ren F., Yu X., Dang W., Niu W., Zhou T., Lin Y., Wu Z., Lin L., Zhong B., Chu H. (2019). Depressive symptoms in Chinese assembly-line migrant workers: A case study in the shoe-making industry. Asia Pac. Psychiatry.

[3] Zhong B.L., Liu T.B., Chan S.S.M., Jin D., Hu C.Y., Dai J., Chiu H.F.K. (2018). Common mental health problems in rural-to-urban migrant workers in Shenzhen, China: Prevalence and risk factors. Epidemiol. Psychiatr. Sci..

[4] Yang M., Dijst M., Helbich M. (2018). Mental Health among Migrants in Shenzhen, China: Does it Matter Whether the Migrant Population is Identified by Hukou or Birthplace?. Int. J. Environ. Res. Public Health.

[5] Durkheim E. (2005). Suicide: A Study in Sociology.

[6] Prati G., Pietrantoni L. (2010). The relation of perceived and received social support to mental health among first responders: A meta-analytic review. J. Community Psychol..

[7] Bruhn J. (2009). The Group Effect: Social Cohesion and Health Outcomes.

[8] Levy J.A., Pescosolido B.A. (2002). Social Networks and Health.

[9] Zhang L. (2001). Strangers in the City: Reconfigurations of Space, Power, and Social Networks within Chinas Floating Population.

[10] Jacka T. (2006). Rural Women in Urban China: Gender, Migration, And Social Change.

[11] Zhou D. (2000). Migrant labor and the ‘dual community’ in the pearl river delta. J. Sun Yatsen Univ. (Soc. Sci. Ed.).

[12] Tong X., Ma X. (2008). ‘Befriending the other’ and ‘breaking up the whole into parts’: The community integration of rural-urban migrants. Soc. Sci. Res..

[13] Nie W., Feng X. (2013). The urban inclusion of migrant workers and their mental health: Based on a survey in the Zhujiang delta area. J. Nanjing Agric. Univ. (Soc. Sci. Ed.).

[14] Yue Z., Li S., Jin X., Feldman M.W. (2013). The Role of Social Networks in the Integration of Chinese Rural–Urban Migrants: A Migrant–Resident Tie Perspective. Urban Stud..

[15] Bian Y. (2004). Source and Functions of Urbanites’ Social Capital: A Network Approach. Soc. Sci. China.

[16] Guan M. (2017). Measuring the effects of socioeconomic factors on mental health among migrants in urban China: A multiple indicators multiple causes model. Int. J. Ment. Health Syst..

[17] Li J., Liu Z. (2018). Housing stress and mental health of migrant populations in urban China. Cities.

[18] Wong D.F.K., Chang Y.-L. (2010). Mental Health of Chinese Migrant Workers in Factories in Shenzhen, China: Effects of Migration Stress and Social Competence. Soc. Work Ment. Health.

[19] Chen W., Zhang Q., Renzaho A.M.N., Ling L. (2019). The Disparity in Mental Health Between Two Generations of Internal Migrants (IMs) in China: Evidence from A Nationwide Cross-Sectional Study. Int. J. Environ. Res. Public Health.

[20] Meng X., Xue S. (2019). Social networks and mental health outcomes: Chinese rural–urban migrant experience. J. Popul. Econ..

[21] Wu M., Duan C., Zhu X. (2016). Effect of social support on psychological well-being in elder rural-urban migrants. Popul. J. (China).

[22] Rook K.S. (1990). Parallels in the Study of Social Support and Social Strain. J. Soc. Clin. Psychol..

[23] Thoits P.A. (2011). Mechanisms linking social ties and support to physical and mental health. J. Health Soc. Behav..

[24] Lin N., Westcott J., Eckenrode J. (1991). Marital Engagement/Disengagement, Social Networks, and Mental Health. The Social Context of Coping.

[25] Cohen S., Wills T.A. (1985). Stress, Social Support, and the Buffering Hypothesis. Psychol. Bull..

[26] Granovetter M.S. (1973). The strength of weak ties. Am. J. Sociol..

[27] Wright K.B., Rains S., Banas J. (2010). Weak-Tie Support Network Preference and Perceived Life Stress Among Participants in Health-Related, Computer-Mediated Support Groups. J. Comput. -Mediat. Commun..

[28] Kawachi I., Berkman L.F. (2001). Social ties and mental health. J. Urban Health Bull. N. Y. Acad. Med..

[29] Stroebe M., Stroebe W., Schut H. (2001). Gender differences in adjustment to bereavement: An empirical and theoretical review. Rev. Gen. Psychol..

[30] Dressler W.W. (1985). Extended family relationships, social support, and mental health in a southern black community. J. Health Soc. Behav..

[31] Dressler W.W., Badger L.W. (1985). Epidemiology of depressive symptoms in black communities. J. Nerv. Ment. Dis..

[32] Beijing Municipal Bureau of Statistics and NBS Survey Office in Beijing Beijing Statistical Yearbook 2020. http://nj.tjj.beijing.gov.cn/nj/main/2020-tjnj/zk/indexch.htm.

[33] Romppel M., Braehler E., Roth M., Glaesmer H. (2013). What is the General Health Questionnaire-12 assessing? Dimensionality and psychometric properties of the General Health Questionnaire-12 in a large scale German population sample. Compr. Psychiatry.

[34] Zhang Y., Cui L., Li K., Jiang Q., Sun X., Gao L., Han Y. (2008). The application of supplemental General Health Questionnaire in the epidemiology of mental illness. Chin. Ment. Health J..

[35] Goldberg D.P., Gater R., Sartorius N., Ustun T.B., Piccinelli M., Gureje O., Rutter C. (1997). The validity of two versions of the GHQ in the WHO study of mental illness in general health care. Psychol. Med..

[36] Wang W., Ding L., Liao Z., Wen C., Hong X., Chen Y., Wang Y. (2012). The best thresholds and the screening features among the three scoring methods of the 12-Item General Health Questionnaire. Chin. J. Psychiatry.

[37] Zhao Y., Luo J. (2005). How to measure social capital: A review of empirical research. Soc. Sci. Abroad (China).

[38] Zhao Y. (2008). Social network and the physical and mental health of urban and rural residents. Soceity (China).

[39] Zhen Y., Zhang Y., Zhu R. (2015). The influence of social support on the mental health of new generation migrants. Dev. Stud. (China).

[40] Lu C., Wu A. (2019). Income gap, social support and mental health of new generation migrant workers. Popul. Dev. (China).

[41] Uchino B.N. (2009). Understanding the Links between Social Support and Physical Health: A Life-Span Perspective with Emphasis on the Separability of Perceived and Received Support. Perspect. Psychol. Sci..

[42] Uchino B.N. (2004). Social Support and Physical Health Understanding the Health Consequences of Relationships.

[43] Milner A., Krnjacki L., LaMontagne A.D. (2016). Age and gender differences in the influence of social support on mental health: A longitudinal fixed-effects analysis using 13 annual waves of the HILDA cohort. Public Health.

[44] Lin N., Ye X., Ensel W.M. (1999). Social Support and Depressed Mood: A structural analysis. J. Health Soc. Behav..

[45] Vandervoort D.J., Skorikov V.B. (2002). Physical health and social network characteristics as determinants of mental health across cultures. Curr. Psychol..

